# Transitions of 24‐H Movement Behaviour Profiles From Schooldays to Weekends and Their Associations With Health‐Related Quality of Life and Well‐Being in Czech Adolescents

**DOI:** 10.1111/cch.70121

**Published:** 2025-06-27

**Authors:** David Janda, Jan Dygrýn, František Chmelík, Lukáš Rubín, Tereza Juřicová, Michal Vorlíček, Ondřej Vencálek, Aleš Gába

**Affiliations:** ^1^ Faculty of Physical Culture Palacký University Olomouc Olomouc Czech Republic; ^2^ Faculty of Science, Humanities and Education Technical University of Liberec Liberec Czech Republic; ^3^ Faculty of Science Palacký University Olomouc Olomouc Czech Republic

**Keywords:** accelerometry, latent class analysis, physical activity, sedentary behaviour, sleep

## Abstract

**Background:**

Adolescents' movement behaviours (MB) vary between schooldays and weekends, potentially impacting health‐related quality of life (HRQoL) and well‐being. This study aimed to identify transitions between 24‐h MB profiles on schooldays and weekends and examine their associations with HRQoL and well‐being.

**Methods:**

This is a cross‐sectional study of 1070 Czech adolescents (average age: 13.8 years and standard deviation: 2.2 years; 56% girls). Participants wore accelerometers for 7 consecutive days to assess physical activity (PA) of different intensities, sedentary behaviour (SB) and sleep. A subsample of 451 participants provided data on HRQoL, which was measured using the Paediatric Quality of Life Inventory, and 484 provided valid well‐being data measured with the 5‐item World Health Organisation Well‐Being Index. Latent transition analysis was used on the MB variables to identify transitions across MB profiles, and linear regression was used to examine associations between transitions and HRQoL or well‐being.

**Results:**

Four MB profiles were identified: *Excellent* (high PA, low SB and high sleep duration), *Good* (average MB values), *Fair* (below‐average PA and sleep, above‐average SB) and *Poor* (low PA and sleep, high SB). Most adolescents transitioned to less favourable profiles on weekends. Those remaining in the *Excellent* profile had higher HRQoL than those transitioning to less favourable profiles. Transitions to the *Poor* profile were associated with the lowest HRQoL and well‐being scores.

**Conclusion:**

This study underscores the dynamic nature of adolescents' MB and the importance of consistent, healthy routines. Interventions optimizing 24‐h MB throughout the week and especially on weekends may enhance adolescent HRQoL and well‐being, but further evidence from longitudinal and intervention studies is needed.

**Summary:**

- We observed a contrast in 24‐h MB between schooldays and weekends: 29.7% of adolescents were in the *Excellent* on schooldays, but only 5.8% did so on weekends, while the prevalence of the *Poor* profile rose from 1.6% on schooldays to 27.7% on weekends.
- Adolescents who maintained the *Excellent* profile across the whole week recorded the highest scores for HRQoL and well‐being.
- Moving into the *Poor* profile on weekend was associated with about 9 points poorer HRQoL and 14 points lower well‐being, compared with peers who remained in the *Excellent* profile.
- Behaviour change strategies should target the entire week to preserve PA, reduce SB and protect sleep.

## 1 Introduction

Movement behaviours (MB) fluctuate considerably between less structured (e.g., weekends or holidays) and more structured days (e.g., schooldays), which are occupied with various obligations such as transport to school, physical education and after‐school programs (Zosel et al. 2022). Differences in the amount of time spent in physical activity (PA), sedentary behaviour (SB) and sleep collectively reported as 24‐h MB, between school and weekend days exemplify such a behavioural change. Specifically, a decrease in PA (Brazendale et al. 2021) and an increase in SB (Fairclough et al. 2015) have been observed among adolescents on weekends compared to schooldays. On the other hand, sleep duration tends to be longer on weekends (Berry et al. 2021). These changes in MB of adolescents are primarily attributed to the higher autonomy on less structured days (i.e., weekends) (Brazendale et al. 2017) allowing them to spend their time more freely in comparison with more structured schooldays.

Research has shown that a high level of SB, such as prolonged sitting or screen time, is associated with negative health outcomes (Carson et al. 2016), including mental disorders (Zhang et al. 2022). In contrast, PA and optimal sleep duration have been consistently shown to have protective effects on both physical (Poitras et al. 2016; Matricciani et al. 2019) and mental health (Marconcin et al. 2022; Short et al. 2019) in adolescents. The recognition of the influence of 24‐h MB on the health (Saunders et al. 2016) and well‐being (Groves et al. 2024) of adolescents has led to a growing body of research aimed at understanding the complex interplay between PA, SB and sleep (Dumuid et al. 2022). Previous studies have identified that MB profiles characterised by high PA, low SB and sufficient sleep are associated with favourable health‐related quality of life (HRQoL) and well‐being in adolescents (Dumuid et al. 2017; de Mello et al. 2024). However, these studies have primarily focused on ‘static’ profiles, with limited evidence on how adolescents change their behavioural patterns across the week.

Even though evidence exists on the associations of 24‐h MB with HRQoL and well‐being (Fairclough et al. 2023; Xiong et al. 2022), most studies integrated a variable‐oriented approach, which limits our understanding of how 24‐h MB patterns are associated with HRQoL and well‐being. Therefore, adopting a person‐oriented approach that acknowledges the heterogeneity of individuals' MB through the identification of distinct behavioural profiles, referred to as typologies (Parker et al. 2019) or patterns (Liu et al. 2019), can enrich our understanding of how these behaviours influence health. Unlike variable‐oriented methods, which examine associations between individual behaviours and outcomes in isolation, person‐oriented approaches capture the real‐world combinations of behaviours that co‐occur within individuals, providing more nuanced insights into MB patterns. For example, Engberg et al. (2022) used a latent class approach to identify behavioural patterns based on digital media use and PA, revealing links to adolescent mental health outcomes. However, understanding the transitions between the MB profiles over the course of a week can provide valuable insights into how daily routines are associated with the HRQoL and well‐being of the young population. To address this research gap, the present study aimed to identify the profiles of 24‐h MB on both schooldays and weekends and the transitions between them. Furthermore, our aim is to investigate the associations between transitions of MB profiles throughout the week and HRQoL and well‐being in adolescents.

## 2 Methods

### 2.1 Participants

The participants in this cross‐sectional study were 1584 adolescents between the ages of 10 and 19 years. They were recruited from 23 elementary and secondary schools in the Czech Republic. Only schools with standard teaching schedules (i.e., same number of physical education classes) were included. Data were collected in the spring or autumn in the two data collection periods, which were joined together (Period 1: 2018–2019, *n* = 824 and Period 2: 2021–2024, *n* = 760). Participants were invited to participate if they were apparently healthy and had no known medical conditions that would affect their ability to participate in 24‐h MB, which was determined by an experienced field researcher.

### 2.2 24‐H MB

Participants were instructed to wear a tri‐axial accelerometer ActiGraph wGT3X‐BT or GT9X Link (ActiGraph LLC, Florida, USA) on their nondominant wrist, capturing raw signal data 24 h a day for 7 consecutive days, excluding activities that involved submerging the device in water for a prolonged period. Devices were initialized to collect tri‐axial acceleration data at 100 Hz. Recordings were downloaded as raw .gt3x files using ActiLife software (v 6.13.3) and analysed with the R package GGIR (v 3.1.1). We used default settings for raw‐data autocalibration (using local gravity as reference), nonwear time detection (via overlapping 60‐min windows shifted every 15 min, with a 15 min epoch labelled nonwear if any window had at least two axes with SD < 13 m*g* and range < 50 m*g*) and imputation of missing or abnormally high values. The Euclidean Norm minus 1 g was used to calculate the average magnitude of dynamic acceleration expressed in milli‐gravitational units (m*g*) (van Hees et al. 2013). Previously published cut‐off points were used to categorise the intensity of the 24‐h MB of participants, i.e., SB (< 35.6 m*g*), light PA (35.6–201.4 m*g*), moderate PA (201.4–707 m*g*) and vigorous PA (> 707 m*g*) (Hildebrand et al. 2017; Hildebrand et al. 2014). The default setting using the Heuristic algorithm (van Hees et al. 2018) looking at the Distribution of Change in Z‐Angle was used to detect sleep time, defined as the duration between sleep onset and awakening.

Participants' day‐level data were excluded if they did not meet the wear‐time criteria, which were sleep duration < 180 min, wake time duration < 240 min, total wear time < 960 min and sleep efficiency < 0.5. These cut‐offs align with the most commonly used criteria (Rodrigues et al. 2025), ensure near‐complete daily coverage, exclude implausibly short sleep or wake periods and minimise misclassification of sleep as SB. All exclusions were then visually inspected and confirmed independently by two researchers (JD and DJ). Participants' 24‐h MB were calculated separately for each day and then averaged separately for schooldays and for weekends. For the analyses, we only included the participants who met the wear‐time criteria for at least 5 valid days, including at least 3 schooldays and both weekend days. After exclusion, the final analytical sample consisted of 1070 individuals.

### 2.3 Well‐Being and HRQoL

Data on HRQoL and well‐being were self‐reported and were collected during the Period 2 data collection. In total, 484 (64% valid data from the Period 2) individuals provided valid well‐being data and 451 (59% valid data from the Period 2) also provided valid HRQoL data. These data were used for the regression analyses examining associations between MB profile transitions and health outcomes.

HRQoL was evaluated using the Paediatric Quality of Life Inventory Version 4.0 (PedsQL). The PedsQL is a validated questionnaire that assesses HRQoL in children and adolescents (Varni et al. 2003). It consists of four domains: physical, emotional, social and school functioning. The items in each domain were scored on a scale from 0 (*Never*) to 4 (*Almost always*) and linearly transformed to 0 to 100 scales, so a higher score indicates better HRQoL. The psychosocial functioning was calculated as an average of items in the emotional, social and school functioning domains. The physical functioning was calculated as an average of items in the physical functioning domain of the PedsQL. The total HRQoL score was calculated as the average of all items in the four domains. In the case of missing data (*n* = 4, 0.9%) in an item in a domain of PedsQL, the mean value over items in that domain was imputed (Fairclough 2010).

Well‐being was assessed using the 5‐item World Health Organisation Well‐Being Index (WHO‐5) (Topp et al. 2015). The WHO‐5 is a widely used questionnaire that evaluates subjective well‐being based on five items related to positive mood, vitality, calmness, self‐esteem and general interest. The items are scored on a scale from 0 (*At no time*) to 5 (*All of the time*), with higher scores indicating higher levels of well‐being. The sum score of the five items multiplied by 4 provides an overall measure of well‐being (on a scale from 0 to 100).

### 2.4 Confounders

Several potential confounders were considered when identifying profiles of 24‐h MB and their transitions, as well as controlling for associations between the transitions of 24‐h MB profiles and HRQoL and well‐being. These confounders included age, sex, body mass index (BMI) *z*‐score and maternal education, selected based on previous literature linking these variables to self‐rated health outcomes in adolescents (von Rueden et al. 2006; Ul‐Haq et al. 2013; Michel et al. 2009). Age and sex were self‐reported. Participants also self‐reported their body height and body weight, from which the BMI *z*‐score was calculated using the World Health Organization's growth reference standards (de Onís et al. 2007).

A questionnaire was administered to the participants to be handed to their parents to collect parental and family characteristics including maternal education. Maternal education was assessed using a question in which mothers indicated the highest level of education completed. Maternal education was dichotomized as low education (up to high school) and high education (university or higher).

Due to missing data in covariates, in BMI *z*‐score (*n* = 54; 5%) and maternal education (*n* = 160; 15%), an imputation algorithm was used to handle missing values under the assumption that data were missing completely at random. The multiple imputation approach (van Buuren and Groothuis‐Oudshoorn 2011) with default setting, using predictive mean matching for BMI *z*‐score and polytomous logistic regression for maternal education, was used.

### 2.5 Statistical Analysis

The statistical analysis for this study was performed using R Statistical Software version 4.3.1 (R Core Team 2021) in the environment of Visual Studio Code version 1.92.0 (Microsoft Corporation, Redmond, USA) and the LatentGold software version 6.0.0 (Statistical Innovations, Arlington, USA). The level of significance was set at 0.05.

#### 2.5.1 MB Profiles

The analytical sample (*n* = 1070) was used to identify profiles of 24‐h MB. First, compositional data analysis was used to deal with the compositional nature of 24‐h MB data, where the time spent in one behaviour necessarily constrains the time available for other behaviours (Dumuid et al. 2020). Compositional data analysis allows for proper interpretation of such data by overcoming issues related to multicollinearity and scale invariance. The time spent in vigorous PA, moderate PA, light PA, SB and sleep was expressed as a set of isometric log‐ratios (*ilr*), using the *compositions* package (Van Der Boogaart and Tolosana‐Delgado 2022).

Second, the *ilrs* on two time points (i.e., schooldays and weekends) were used in latent transition analysis using LatentGold software (Statistical Innovations, Arlington, USA). A latent transition model is a type of hidden Markov model that allows for the identification of latent states and transitions between them over time (Collins and Lanza 2009). The model assumes an underlying structure in the data, allowing for the identification of MB profiles on schooldays and weekends and how individuals transition between them. Covariances of the *ilrs* were included in the model as *ilrs* are used to construct a coordinate system with a regular covariance matrix and different *ilr* coordinate systems are just mutual rotations. Additionally, we have used the least restricted models, allowing for variance and covariance to differ across latent states, as well as to differ across time. The fit of the latent transition models was assessed using model fit indices (e.g., Bayesian information criterion and Akaike information criterion), where lower values indicate better fit. Entropy was used to verify the level of certainty in the separation of profiles, higher values indicating better separation. Additionally, we have evaluated the identified profiles by their practical relevance and size (Collins and Lanza 2009).

The presence of rounded zero values in 24‐h MB data does not allow the application of compositional data analysis as *ilr* that includes a zero cannot be computed (Rasmussen et al. 2020). Our dataset included 34 observations with zero values in vigorous PA; because of that, the zero values were imputed using the log‐ratio expectation–maximisation algorithm (Rasmussen et al. 2020). These imputations led to the identification of a profile that consisted of less than 2% of the total dataset in solutions of 3–5 profiles. Due to this finding, we have decided to omit the observations with zero values from our dataset.

Finally, after identifying the best model, the modal assignment was used to assign each participant to the profile on schooldays and weekends for which they had the highest posterior probability. The profiles were labelled based on values of the overall 24‐h MB composition to facilitate better presentation. Descriptive statistics for each profile were calculated as weighted means and standard deviations (SD) or weighted proportions, using the posterior probability of assignment to a given profile as a weight for each observation.

#### 2.5.2 Associations Between Transitions of MB Profiles and HRQoL and Well‐Being

Based on the results of the latent transition analysis, we have created categories of transitions between the identified profiles from schooldays to weekends. Individuals with transitions occurring at a frequency of less than 1% were excluded from the analysis due to insufficient sample size for meaningful interpretation. Consequently, linear regression models were used to investigate the associations between the transitions between MB profiles and HRQoL and well‐being. Three separate models were built in case of HRQoL, focusing on the psychosocial functioning, physical functioning and the overall score of HRQoL. One model was created for the overall score of well‐being. All models were adjusted for age, sex, BMI *z*‐score and maternal education.

## 3 Results

### 3.1 Sample Description

The descriptive characteristics of the study participants are shown in Table 1. The mean age of the participants was 13.8 ± 2.2 SD years, from which more than half were girls (56%), with an average BMI z‐score of 0.22 ± 1.12 SD. More than 41% of individuals had mothers with higher education. On average, participants spent more time on schooldays in vigorous PA by 1.8 min (4.0 vs. 2.2 min), by 9.1 min in moderate PA (42.1 vs. 33.0 min), by 9.5 min in light PA (274.9 vs. 265.3 min) and by 17.1 min in SB (634.2 vs. 617.1 min) but spent less time in sleep by 67.6 min (484.9 vs. 552.5 min) in comparison with weekends (Table 2). The overall HRQoL score was 75.0 ± 11.6 SD points, while the psychosocial and physical functioning scores were 70.9 ± 13.4 SD and 82.8 ± 11.3 SD points, respectively. The average well‐being score was 59.0 ± 18.9 points.

**TABLE 1:** Descriptive characteristics of total sample and of the identified profiles (*n* = 1070).

	Total sample		Excellent		Good		Fair		Poor	
	Mean	SD	Mean	SD	Mean	SD	Mean	SD	Mean	SD
**Schooldays (%)** ^**a**^			**29.7**		**43.0**		**25.8**		**1.6**	
Age, years	13.8	2.2	12.2	1.6	13.9	1.9	15.4	1.9	16.4	1.7
Girls (%)	55.6		54.0		55.6		58.1		43.8	
BMI *z*‐score	0.22	1.12	0.13	1.16	0.26	1.08	0.25	1.09	0.16	1.12
Maternal higher education (%)	41.6		40.8		42.7		41.2		33.4	
**HRQoL (*n* = 451)**										
Overall score	75.0	11.6	79.0	10.4	74.6	11.0	71.0	12.6	71.3	13.1
Psychosocial functioning	70.9	13.4	75.4	12.1	70.5	12.8	66.4	14.5	66.3	14.3
Physical functioning	82.8	11.3	85.9	10.0	82.4	11.1	79.8	12.1	80.7	12.6
**Well‐being score (*n* = 484)**	59.0	18.9	65.0	17.3	58.7	18.3	53.3	19.5	55.0	21.5
**Weekends (%)** ^**a**^			**5.8**		**24.3**		**42.2**		**27.7**	
Age, years	13.8	2.2	12.0	1.4	12.3	1.7	13.9	2.0	15.4	2.0
Girls (%)	55.6		54.4		54.1		56.1		56.4	
BMI *z*‐score	0.22	1.12	0.10	1.14	0.14	1.17	0.26	1.08	0.24	1.09
Maternal higher education (%)	41.6		50.3		38.5		42.9		40.4	
**HRQoL (*n* = 451)**										
Overall score	75.0	11.6	81.1	10.6	78.6	10.4	74.7	11.0	71.1	12.5
Psychosocial functioning	70.9	13.4	77.8	12.1	74.9	12.1	70.5	12.8	66.4	14.4
Physical functioning	82.8	11.3	87.5	10.6	85.5	9.9	82.4	11.1	79.9	12.0
**Well‐being score (*n* = 484)**	59.0	18.9	69.1	16.5	64.2	17.4	58.6	18.3	53.7	19.5

Profile description: *Excellent—*High physical activity, low sedentary behaviour, high sleep duration. *Good—*Above average of all movement behaviours values. *Fair—*Below average physical activity and sleep, above average sedentary behaviour. *Poor—*Low physical activity and sleep, high sedentary behaviour.

Abbreviations: BMI = Body Mass Index, HRQoL = Health‐Related Quality of Life, SD = Standard Deviation.

^a^ Proportion of individuals in each profile.

**TABLE 2:** 24‐h movement behaviours of total sample and of the identified profiles on schooldays and weekends (*n* = 1070).

	Total sample	Excellent	Good	Fair	Poor
	Mean^a^	Mean^a^	Mean^a^	Mean^a^	Mean^a^
**Schooldays (%)** ^**b**^		**29.7**	**43.0**	**25.8**	**1.6**
VPA (min/day)	4.0	7.6	3.7	1.1	0.3
MPA (min/day)	42.1	57.0	41.2	24.8	11.9
LPA (min/day)	274.9	313.1	267.5	229.7	183.3
SB (min/day)	634.2	556.6	647.7	734.2	838.6
Sleep (min/day)	484.9	505.7	479.9	450.3	406.0
**Weekends (%)** ^**b**^		**5.8**	**24.3**	**42.2**	**27.7**
VPA (min/day)	2.2	12.1	6.0	1.8	0.4
MPA (min/day)	33.0	72.6	53.6	32.9	16.1
LPA (min/day)	265.3	342.3	298.6	261.6	213.7
SB (min/day)	617.1	455.6	541.4	626.3	732.2
Sleep (min/day)	552.5	557.5	540.4	517.4	477.5

Abbreviations: LPA = Light Physical Activity, MPA = Moderate Physical Activity, SB = Sedentary Behaviour, VPA = Vigorous Physical Activity.

^a^ Compositional mean for each movement behaviour.

^b^ Proportion of individuals in each profile.

### 3.2 24‐H MB Profiles

The model selection criteria for the latent transition models with 1 to 5 profiles are presented in Table 3. The information criteria, meaning and average size of profiles indicated best fit in model for 4 profiles across schooldays and weekends. On both schooldays and weekends, identified profiles (Table 2) included individuals with longest time spent in PA of all intensities, above average sleep duration and lowest SB who were labelled as *Excellent* (30% on schooldays and 6% on weekends), followed by individuals with average values in all 24‐h MB who were labelled as *Good* (43% on schooldays and 24% on weekends). Individuals with below average values of PA indicators and sleep, but above average levels of SB, were labelled as *Fair* (26% on schooldays and 42% on weekends), followed by individuals with shortest time spent in PA of all intensities, shortest sleep duration and most time in SB who were labelled as *Poor* (2% on schooldays and 27.7% on weekends). All profiles showed a similar change of 24‐h MB composition between school and weekend days, resulting in increased time spent in PA of all intensities as well as sleep, but decrease in SB duration.

**TABLE 3:** Statistical indicators for latent transition models with 2–5 profiles (*n* = 1070).

	2 profiles	3 profiles	4 profiles	5 profiles
BIC	5383	5173	**5115**	5127
AIC	5209	4905	**4742**	4640
Entropy	0.69	0.69	**0.72**	0.70
Minimal size (%)^a^	47.0	23.7	**14.7**	10.1

*Note:* Boldface values indicate best fit based on our selection criteria.

Abbreviations: AIC = Akaike information criterion, BIC = Bayesian information criterion.

^a^ Minimal size reflects the minimum size of the profiles in each solution averaged across schooldays and weekends.

### 3.3 Transitions Between Identified Profiles

The transition probabilities estimated from the total sample indicated that most individuals transitioned to the profiles with lower PA values of all intensities, but more sleep (Table 4). Most individuals (80%) in the *Excellent* profile transitioned to the *Good* profile, while 19.6% remained stable. Most individuals (95%) in the *Good* profile transitioned to the *Fair* and almost 4% to the *Poor* profile. Similar proportions transitioned from *Fair* to *Poor* (95%) and less than 5% remained stable. The *Poor* profile was the most stable as only less than 8% transitioned to other profiles, with most transitioning (7%) to the *Good* profile.

**TABLE 4:** Transition probabilities between the identified typologies.

Profile	Excellent	Good	Fair	Poor
**Proportion schooldays (%)**	29.7	43.0	25.8	1.6
**Transition probabilities**	**From Excellent**	**From Good**	**From Fair**	**From Poor**
To Excellent (%)	19.6	0.0	0.0	0.4
To Good (%)	80.0	0.8	0.1	6.5
To Fair (%)	0.1	95.3	4.7	0.9
To Poor (%)	0.3	3.9	95.2	92.2
**Proportion weekends (%)**	5.8	24.3	42.2	27.7

Profile description: *Excellent—*High physical activity, low sedentary behaviour, high sleep duration. *Good—*Above average of all movement behaviours values. *Fair—*Below average physical activity and sleep, above average sedentary behaviour. *Poor—*Low physical activity and sleep, high sedentary behaviour.

### 3.4 Associations Between Identified Transitions and HRQoL and Well‐Being

The observed transitions between the profiles from schooldays to weekends included the following: from *Excellent* to *Excellent*, from *Excellent* to *Good*, from *Good* to *Fair* and from *Fair* or *Poor* to *Poor*. The *Fair* and *Poor* profiles on schooldays were joined due to low frequency in the *Poor* profile. Table 5 displays the associations between the observed transitions and HRQoL and well‐being. Compared to the transition from *Excellent* to *Excellent*, the transition from *Good* to *Fair* was associated with a lower HRQoL score by 6.4 (95% confidence interval [CI] = −12.4; −0.3, *p* = 0.040) and a lower psychosocial functioning by 7.4 (95% CI = −14.6, −0.4; *p* = 0.040). The transition from *Fair* or *Poor* to *Poor* was associated with overall HRQoL score lower by 9.5 (95% CI = −15.9, −3.2; *p* = 0.003), psychosocial functioning lower by 10.8 (95% CI = −18.2, −3.3; *p* = 0.005), physical functioning lower by 7.2 (95% CI = −13.5, −0.9; *p* = 0.025) and well‐being score lower by 14.4 (95% CI = −24.9, −3.9; *p* = 0.007) in comparison with the transition from *Excellent* to *Excellent*.

**TABLE 5:** Associations between the transitions of movement behaviour profiles from schooldays to weekends and HRQoL (*n* = 451) and well‐being (*n* = 484).

	Overall HRQoL score			Physical functioning			Psychosocial functioning			Well‐being score		
	B	95% CI	*p*‐value	B	95% CI	*p‐*value	B	95% CI	*p*‐value	B	95% CI	*p*‐value
*Excellent* to *Excellent*	*Reference*			*Reference*			*Reference*			*Reference*		
*Excellent* to *Good*	−2.5	−8.5, 3.6	0.425	−0.9	−7.0, 5.1	0.758	−3.3	−10.4, 3.8	0.366	−5.1	−15.2, 5.0	0.320
*Good* to *Fair*	**−6.4**	**−12.4, −0.3**	**0.040**	−4.3	−10.3, 1.7	0.163	**−7.4**	**−14.6, −0.4**	**0.040**	−9.8	−19.8, 0.3	0.056
*Fair* or *Poor* to *Poor*	**−9.5**	**−15.9, −3.2**	**0.003**	**−7.2**	**−13.5, −0.9**	**0.025**	**−10.8**	**−18.2, −3.3**	**0.005**	**−14.4**	**−24.9, −3.9**	**0.007**

*Note:* All models were adjusted for sex, age, Body Mass Index *z*‐score and maternal education level. Boldface values indicate significant results at *p* < 0.05.

Abbreviations: *B* = Unstandardised regression coefficient, CI = Confidence interval, HRQoL = Health‐related quality of life.

## 4 Discussion

To better understand how adolescents change their MB from schooldays to weekends, we utilised a person‐oriented approach on 24‐h accelerometer‐derived MB data to identify transitions between MB profiles on schooldays and weekends. Additionally, we have investigated how the transitions between MB profiles on schooldays and weekends are associated with indicators of HRQoL and well‐being. Four distinct profiles were identified on schooldays and weekends that differ in the 24‐h MB. Representing a shift toward less favourable profiles, most adolescents showed a reduction in PA of all intensities and SB, and an increase in sleep duration on weekends. Our results indicate that adolescents who maintained the *Excellent* profile from schooldays to weekends were associated with higher HRQoL and well‐being scores in comparison with other individuals.

Our findings align with previous research indicating that adolescents tend to be less physically active (Brazendale et al. 2021) and less sedentary (Burchartz et al. 2022), but sleep more (Berry et al. 2021) on weekends compared to schooldays. The observed increase in sleep duration on weekends, coupled with lower levels of PA of all intensities and SB, reflects a shift in daily structure. The reduction in PA across all intensities was consistent with prior research (Brazendale et al. 2021), as absolute change was approximately 10 min of moderate‐to‐vigorous PA, which could contribute to a lower likelihood of achieving the recommended 60 min of moderate‐to‐vigorous PA per day (Bull et al. 2020). The shift in sleep duration may be caused by social jetlag (i.e., the discrepancy between biological and social rhythms) and sleep deprivation leading to catch‐up sleep on weekends (Tonetti et al. 2022). Especially, individuals who remained in the *Poor* profile had longer sleep on weekends by 72 min, which may be one of the contributing factors to lower well‐being as it was shown before that longer sleep catch‐up may lead to a lower well‐being (Tonetti et al. 2022). Contrarily, it was suggested that an increase in sleep duration and time spent in moderate‐to‐vigorous PA at the expense of other behaviours may increase HRQoL score (Sampasa‐Kanyinga et al. 2021) and decrease symptoms of depression (Tan et al. 2023). Suggesting that individuals in the least favourable profiles, i.e., *Poor* would benefit the most from targeted interventions promoting increased sleep duration on schooldays, while increasing overall PA during the whole week. These results support previous findings that emphasize the need for multidimensional approaches in research and interventions targeting adolescent health, as combinations of MB have been shown to better predict health outcomes than single behaviours alone (Saunders et al. 2016).

The distribution of adolescents across the identified MB profiles differed notably between schooldays and weekends. On schooldays, the most common profiles were *Excellent* and *Good* (29.7% and 43.0%, respectively), reflecting relatively structured and favourable 24‐h MB compositions. On weekends, however, the *Fair* and *Poor* profiles became more prevalent (42.2% and 27.7%), suggesting that many adolescents shifted toward less favourable patterns. These differences may partly reflect the structured nature of weekdays, which include scheduled activities such as commuting, physical education lessons and consistent school routines that encourage regular sleep and PA. In contrast, weekends offer more autonomy and unstructured time, which may lead to later bedtimes and reduced PA. This trend is described by the Structured Day Hypothesis (Brazendale et al. 2021; Brazendale et al. 2017), which posits that unstructured days such as weekends lead to a decrease in PA and an overall change in behaviour. Similarly, a study on German children and adolescents (Burchartz et al. 2022) showed that on weekends, adolescents tend to sleep longer, leaving less time for PA and SB. These findings underscore the importance of studying MB across different segments of the week, as transitions between structured and unstructured days may meaningfully impact adolescents' overall MB profiles and related health outcomes.

We identified only a small proportion of individuals who stayed in the same profile when transitioning from schooldays to weekends. Specifically, the most active and least sedentary individuals in the *Excellent* profile (20%) and the least active and most sedentary individuals in the *Poor* profile (92%) exhibited highest stability. This may be due to various reasons, for example, highly active individuals may sustain or increase their activity on weekends due to possible sport activities (i.e., tournaments), while the least active individuals remain inactive as their home environment does not promote PA (McMinn et al. 2013). The increase of PA and reduction of SB of individuals in the *Poor* profile is therefore necessary over the course of the whole week to improve the least favourable 24‐h MB compositions.

Despite that most individuals transition to a different profile, there were not individuals who would change their behaviour extensively (e.g., transition from *Fair* to *Excellent* profile or vice‐versa). These findings suggest that 24‐h MB interventions should target the entire week, with the aim of supporting more adolescents in achieving MB compositions similar to those observed in the *Excellent* or *Good* profiles, which may contribute to improved HRQoL and well‐being. However, most interventions focus on schooldays as schools are more accessible, but lead to minimal improvements in PA (Love et al. 2019), suggesting that including the whole week and targeting individuals with the least favourable 24‐MB may be more beneficial.

The associations of favourable profile (i.e., *Excellent*) during the transition from schooldays to weekends with higher HRQoL and well‐being scores highlight the importance of optimised 24‐h MB during the whole week. Our results are consistent with previous literature suggesting a linear trend between more favourable 24‐h MB composition and better HRQoL or well‐being (Groves et al. 2024; Verswijveren et al. 2024). The physiological benefits of regular PA and optimal sleep were previously linked to lower levels of stress hormones which may lead to higher well‐being score (Mahindru et al. 2023; Chow 2020). However, future longitudinal studies are needed to better understand how the regular behavioural routines contribute to improving well‐being (de Sa Couto‐Pereira et al. 2024).

The strength of our study is the use of person‐oriented approach utilising the latent transition analysis on 24‐h MB data, which allowed for the identification of distinct profiles, providing a nuanced understanding of how adolescents' MB combine together, and how individuals change their behaviour from schooldays to weekends. Additionally, the use of 24‐h raw accelerometer data strengthens the validity and reliability of our findings. The inclusion of participants with valid accelerometer data for at least 3 schooldays and 2 weekend days could be considered a strength as most accelerometer studies relies on the 3 schooldays plus 1 weekend day protocol. Finally, the use of various measures of HRQoL and well‐being indicators allowed us to evaluate individuals' perceived well‐being from different perspectives.

This study has several limitations that should be acknowledged. The cross‐sectional design restricts the ability to establish causal relationships between MB transitions and HRQoL or well‐being, necessitating longitudinal research to better understand these dynamics. Additionally, the use of a simplified measure of well‐being (WHO‐5) may not capture the whole well‐being construct as compared to more complex tools (VanderWeele et al. 2020). Furthermore, while we utilised a person‐oriented approach which enabled us to identify distinct MB profiles based on 24‐h compositions, this method inherently groups individuals with similar patterns. As a result, it may oversimplify the complexity and variability of individual MB, potentially masking important within‐profile differences. Moreover, our sample size did not allow us to identify all possible transitions between the identified MB profiles, and a bigger sample could provide more detailed insights. Finally, our study is limited to the analysis of MB intensities and durations, while the qualitative side of MB, such as posture (e.g., sitting or standing) or sleep quality, may provide additional information.

In conclusion, this study identified four distinct 24‐h MB profiles on schooldays and weekends, suggesting the dynamic nature of adolescents' behaviour. Our findings underscore that most adolescents decrease their PA and SB on weekends but increase sleep duration in comparison with schooldays. The association between more favourable MB profiles and higher HRQoL and well‐being suggests that interventions should aim to promote consistent, healthy routines throughout the week. Future research should focus on understanding the underlying factors driving these behavioural transitions. Additionally, future research should investigate the long‐term implications of transitions between schooldays and weekends in diverse adolescent populations to better understand how consistent fluctuations in MB influence health and well‐being over extended periods.

## Author Contributions

**David Janda:** conceptualization, investigation, writing – original draft, visualization, formal analysis. **Jan Dygrýn:** writing – review and editing, methodology, software, data curation. **František Chmelík:** writing – review and editing, data curation, investigation. **Lukáš Rubín:** writing – review and editing, data curation, investigation. **Tereza Juřicová:** writing – review and editing, data curation, investigation. **Michal Vorlíček:** writing – review and editing, data curation, investigation. **Ondřej Vencálek:** methodology, writing – review and editing, formal analysis, supervision. **Aleš Gába:** writing – review and editing, writing – original draft, supervision, funding acquisition.

## Ethics Statement

The study was conducted following ethical guidelines and approval from the local ethical committee (reg. no. 19/2017 and 80/2021). Informed consent was obtained from the participants' parents or legal guardians prior to their participation in the study.

## Conflicts of Interest

The authors declare no conflicts of interest.

## Data Availability

The dataset is available at https://doi.org/10.5281/zenodo.15631612. The R code and the LatentGold syntax are available at a request from the corresponding author.
